# Spike timing-dependent plasticity under imbalanced excitation and inhibition reduces the complexity of neural activity

**DOI:** 10.3389/fncom.2023.1169288

**Published:** 2023-04-12

**Authors:** Jihoon Park, Yuji Kawai, Minoru Asada

**Affiliations:** ^1^Center for Information and Neural Networks, National Institute of Information and Communications Technology, Suita, Japan; ^2^Symbiotic Intelligent Systems Research Center, Institute for Open and Transdisciplinary Research Initiatives, Osaka University, Suita, Japan; ^3^Chubu University Academy of Emerging Sciences/Center for Mathematical Science and Artificial Intelligence, Chubu University, Kasugai, Japan; ^4^International Professional University of Technology in Osaka, Osaka, Japan

**Keywords:** E/I balance, spiking neural network, complexity, information transmission, neuropsychiatric brain disorder, self-organization

## Abstract

Excitatory and inhibitory neurons are fundamental components of the brain, and healthy neural circuits are well balanced between excitation and inhibition (E/I balance). However, it is not clear how an E/I imbalance affects the self-organization of the network structure and function in general. In this study, we examined how locally altered E/I balance affects neural dynamics such as the connectivity by activity-dependent formation, the complexity (multiscale entropy) of neural activity, and information transmission. In our simulation, a spiking neural network model was used with the spike-timing dependent plasticity rule to explore the above neural dynamics. We controlled the number of inhibitory neurons and the inhibitory synaptic weights in a single neuron group out of multiple neuron groups. The results showed that a locally increased E/I ratio strengthens excitatory connections, reduces the complexity of neural activity, and decreases information transmission between neuron groups in response to an external input. Finally, we argued the relationship between our results and excessive connections and low complexity of brain activity in the neuropsychiatric brain disorders.

## 1. Introduction

The neural network structure and its function are interdependently organized. That is, neural activity emerge through the cycle of interactions between neurons connected by synapses, and the synaptic efficacy changes depending on neural activity. The interaction is influenced by the neural physiology that determines the behavior of an individual neuron and synapse. Thus, abnormalities in the physiological parameters may impair the organization processes of the neural structure and function, leading to altered responses to sensory stimuli.

The excitation and inhibition (E/I) of neurons are the fundamental physiological basis of neural circuits. An atypical E/I balance is considered the main precipitating factor for schizophrenia and autism spectrum disorder (ASD) (Rubenstein and Merzenich, 2003; O'Donnell, 2011; Nelson and Valakh, 2015). Hashemi et al. (2018) reported that the number of inhibitory neurons (in particular, parvalbumin-expressing interneurons) in the prefrontal cortex of patients with ASD was less than that in typical developing (TD) persons, whereas the number of excitatory neurons was comparable. In addition, a study using a mouse model of ASD showed a reduction in inhibitory neurons in only one hemisphere of the parietal and occipital cortices rather than throughout the brain (Gogolla et al., 2009). Further, a decrease in gamma aminobutyric acid (GABA) receptors, which affects the E/I balance, was also reported in the brains of patients with ASD (Fatemi and Folsom, 2009; Oblak et al., 2009, 2011; Blatt and Fatemi, 2011). Furthermore, using optogenetic tools, Yizhar et al. (2011) showed that the elevation of E/I balance, i.e., weaker inhibition, within the medial prefrontal area of mice induced impairments in social behaviors similar to those observed in patients with ASD and impaired information transmission, i.e., a decrease in mutual information between a neuron and a stimulus.

Studies on the brains of individuals with neuropsychiatric disorders have reported low complexity of neural activity (Bosl et al., 2011; Ghanbari et al., 2015; Hadoush et al., 2019; Xu et al., 2020) and discussed its relationship with the E/I balance. Complexity is often evaluated in terms of the unpredictability of time-series signals, and a method that uses sample entropy over multiple time scales [multiscale entropy (MSE)] has been proposed to evaluate the complexity (Costa et al., 2002, 2005). Bosl et al. (2011) measured electroencephalogram (EEG) signals during the resting state in children with ASD and TD and showed that the mean of MSE across channels in the children with ASD was lower than that in the TD children. Further, using magnetoencephalography (MEG), Ghanbari et al. (2015) evaluated children with ASD during the resting state and showed that the complexity of MEG signals differed from that in TD children, depending on the brain region and frequency band. Although the authors hypothesized that the tight regulation of neural activity by GABA might lead to the reduced complexity of neural activity (Ghanbari et al., 2015), the specific mechanism is not well understood.

Various studies have reported an atypical brain structure in individuals with neuropsychiatric disorders such as increased neuronal density of some local regions (cortical areas M1, V1, frontal association cortex, and S1) (Casanova et al., 2006), excessive connections between regions (Solso et al., 2016), and decreased global connectivity in the frontal and temporal regions (Van der Meij and Voytek, 2018). Although these findings are controversial, various studies from different perspectives indicated that these atypical structural characteristics may indicate a disruption in the interaction between brain regions. As a result, characteristic behavior and brain activity indicative of neuropsychiatric disorders are observed. However, it is unclear how an altered E/I balance in a local brain region may affect the low complexity of neural activity and structural characteristics, especially from the perspective of organization of the brain through structure-function interactions.

Several studies have investigated the relationship between the E/I balance and neural activity using a computational model. Most of these studies showed that globally weak or absent inhibitory connections in the network resulted in atypical neural activity, e.g., unstable dynamics (Loh et al., 2007), less information transmission (Deco et al., 2014), reduced neural oscillation (Börgers and Kopell, 2003), and an impairment in the self-organization of the network (Yamada et al., 2013). However, as previously mentioned, physiological abnormalities related to the E/I imbalance were observed in some areas of the brain (Gogolla et al., 2009). Therefore, it is necessary to examine not only the globally atypical inhibition but also the effect of atypical inhibition in a local brain region on the entire brain structure and its activity. Further, synapses in the brain exhibit plasticity. Therefore, synaptic connections between neurons can be modified depending on neural activity, and the altered synapses in turn affect neural activity. To the best of our knowledge, no study has examined how locally altered E/I balance in a single brain region affects both the mutual organization of an atypical network structure and the low complexity of neural activity in the brain.

Park et al. (2019) investigated the relationship between the network structure and the complexity of neural activity using a computational model. They simulated a spiking neural network model consisting of multiple neuron groups with different macroscopic network structures. Their results showed that the complexity of neural activity decreased in a neuron group with many local connections, i.e., local over-connectivity. However, since the global structure among neuron groups was given, it is not clear how the excessive connectivity emerges through the spike timing-dependent plasticity (STDP) that one of the plasticity rules to change synaptic weights based on the spike timing between two neurons.

**Figure 1:**
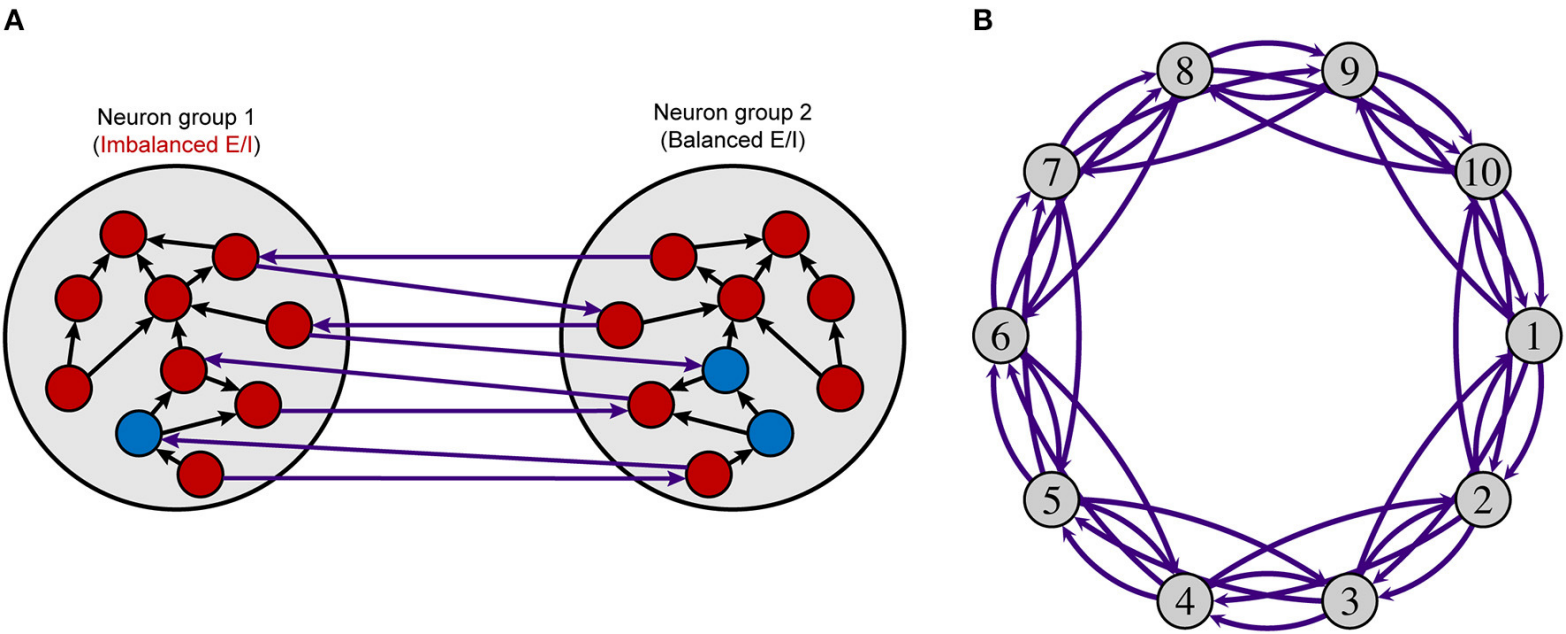
Neuron group model. **(A)** Intra- and interconnections in a model with two neuron groups. **(B)** A network model using ten neuron groups. Each neuron group comprises excitatory (red nodes) and inhibitory (blue nodes) neurons, as shown in **(A)**. Excitatory neurons have intra- (black arrows) and interconnections (purple arrows), and inhibitory neurons have intraconnections only. Each white circle indicates a neuron group and purple arc arrows show connections between neuron groups. The number of inhibitory neurons and the synaptic weights from inhibitory neurons to excitatory neurons in neuron group 1 (imbalanced neuron group) are changed to control the local E/I balance. The parameters in other neuron groups (balanced neuron group) are not changed. E/I, excitation and inhibition.

The main objective of this study is to elucidate how locally altered E/I balance affects not only neural activity and information transmission, but also the organization of connectivity in the brain using a spiking neural network model. We hypothesize that organization through the interaction of the neural structure and function under local E/I imbalance leads to excessive connectivity between neurons and decreased complexity of neural activity, resulting in decreased information transfer in response to an external input. To verify this hypothesis, we constructed a model consisting of multiple spiking neuron groups and controlled the number of inhibitory neurons and the inhibitory synaptic weights to excitatory neurons within a single neuron group to change the E/I balance locally (see Figure 1). Further, we used the STDP to change excitatory synaptic weights in the model. We used the average weights of connections in each neuron group and between neuron groups to evaluate the neural network structure after the STDP process and used MSE to evaluate the complexity of neural activity. To evaluate information transfer in the network in response to an external input, we measured the mutual information (MI) between each neuron group and an external input and the transfer entropy (TE) between neuron groups when external input was given. Finally, we discuss the correspondence between our simulation results and existing studies in neuropsychiatric brain disorders that showed excessive connections and low complexity of brain activity.

## 2. Materials and methods

### 2.1. Network model

Figure 1 shows an overview of the model used in this study. The model consists of multiple neuron groups, each of which has 800 excitatory and *N*_I_ inhibitory neurons. We used regular-spiking neurons and fast-spiking neurons from the Izhikevich model (Izhikevich, 2003, 2006; Izhikevich and Edelman, 2008) for the excitatory and inhibitory neurons, respectively. Here, the fast-spiking neuron corresponds to a parvalbumin-expressing interneuron of which the number and activity are smaller in the brains of individuals with psychiatric than in those of normal healthy persons (Fatemi and Folsom, 2009; Oblak et al., 2009, 2011; Blatt and Fatemi, 2011; Hashemi et al., 2018). Each excitatory neuron has 70 connections with randomly selected neurons within the same neuron group (intraconnection) and 30 connections with randomly chosen neurons in other neuron groups (interconnection). These parameters were determined based on the biological evidence that the ratio of local and global connections of pyramidal excitatory neurons is ~7:3 (Gruner et al., 1974). Intra- and interconnections have time delays for the propagation of potentials sampled from uniform distributions in the range of 2–4 and 4–10 ms, respectively. The synaptic weights of these excitatory connections change based on an STDP rule (Pfister and Gerstner, 2006), which is a biologically plausible plasticity mechanism. Each inhibitory neuron has 100 intraconnections but does not have interconnections. To control the strength of inhibition by parameters, we assume that inhibitory synapse does not have plasticity, i.e., two synaptic weight matrices, *W*_IE_, from inhibitory neurons to excitatory neurons, and *W*_II_, between inhibitory neurons, are fixed. The time delay for inhibitory connections is sampled from a uniform distribution in the range of 1–3 ms.

### 2.2. Simulation setting

We performed simulations using two kinds of network models; one with two neuron groups and the other with ten neuron groups, to investigate the effects of the local E/I imbalance in a single neuron group on the entire network. The following parameters were used to control the E/I balance in a neuron group:

- *N*_I_: Number of inhibitory neurons {100, 150, 200 (baseline), 250, 300}.
- *W*_IE_: Synaptic weights from inhibitory neurons to excitatory neurons {0.0125, 0.01875, 0.025 (baseline), 0.03125, 0.0375}.

Hereafter, a neuron group with these control parameters is called an imbalanced neuron group, while other non-controlled (baseline) neuron groups are called balanced neuron groups. The imbalanced neuron group is shown as neuron group 1 in Figure 1. The parameters in the balanced neuron groups were set to *N*_I_ = 200, *W*_IE_ = 0.025, and *W*_II = 0.013_. Initial excitatory synaptic weight *W*_E_ were sampled from uniform distributions in the range of 0.0–0.04. The number of inhibitory neurons were determined based on the existing study that showed the ratio of number of excitatory and inhibitory neurons in the mammalian cortex is ~4:1 (Nowak et al., 2007). The parameters for synaptic weights were determined to show resting-state-like neural activity *in vivo* (Softky and Koch, 1993; Wilson, 1994), where each neuron shows Poisson firing patterns with low frequency after the STDP process.

The total simulation time was 1,520 s, and one time step was 0.05 ms. The duration for changes of excitatory synaptic weights through STDP was set as 1,500 s from 5 s. Each neuron received excitatory input of homogeneous Poisson spike trains with 0.6 Hz throughout the simulation using the following equation:


(1) $$\begin{array}{l} P\left(n\text{ spikes during Δ}t\right)=e^{-\lambda \text{Δ}t}\frac{{\left(\text{λΔ}t\right)}^{n}}{n!}, \end{array}$$

where, λ is the firing rate, and *n* is the number of spikes during a time interval Δ*t*. A synaptic weight of Poisson input was set to 0.6. After the changes of synaptic weights, the complexity was evaluated using neural activity from 1,510 s for 5 s, when neural activity reached a steady state. Neural activity in each neuron group is represented as a time series of the local average potential (LAP), which is the average membrane potential of excitatory neurons in a neuron group. Therefore, LAP reflects the synchronized neural activity of excitatory neurons. The simulation was conducted independently 20 times for each condition.

To investigate how well the organized network can transfer the external input to other neuron groups, we measured the MI between the external input and neural activity of each neuron group when the external input was given to excitatory neurons in one neuron group in the network from 1,515 s for 5 s. We also measured the TE to understand the information transmission from neural activity of a neuron group receiving external input to those of other neuron groups. In this study, we constructed the external input using an inhomogeneous Poisson process. The firing rate of the process λ was determined by λ(*t*) = 25sin^2^(2π*t*). A synaptic weight of external Poisson input was set to 0.05. The LAP signal in each neuron group and the summation of external Poisson input in each time step were used to calculate the MI and TE.

### 2.3. Neuron model

We used regular-spiking and fast-spiking neurons of the Izhikevich neuron model (Izhikevich, 2003, 2006; Izhikevich and Edelman, 2008). The equations are as follows:


(2) $$\begin{array}{lll} \frac{\text{d}v}{\text{d}t} & = & 0.04v^{2}+5v+140-u+I_{\text{syn}}, \end{array}$$

(3) $$\begin{array}{lll} \frac{\text{d}u}{\text{d}t} & = & a\left(bv-u\right), \end{array}$$

(4) $$\begin{array}{l} \text{if }v\;\geq \;30\text{ mV},\text{ then }\{\begin{array}{l} v\leftarrow c\\ u\leftarrow u+d, \end{array}\; \end{array}$$

where *v* and *u* represent membrane potential and recovery variable, respectively. Variables *a* and *b* denote the time scale and sensitivity of the recovery variable *u*, respectively. Variables *c* and *d* represent the reset values of the membrane potential and the recovery variable after the spike, respectively. These parameters are the same as in other studies (Izhikevich, 2003, 2006). Synaptic current *I*_syn_ is expressed as follows:


(5) $$\begin{array}{l} I_{\text{syn}}=g_{\text{AMPA}}(0-v)+g_{\text{NMDA}}\frac{{\left[(v+80)/60\right]}^{2}}{1+{\left[(v+80)/60\right]}^{2}}(0-v)\;\\ \;+g_{\text{GABA}}(-70-v), \end{array}$$

Each conductance *g* is given by:


(6) $$\begin{array}{l} \frac{\text{d}g}{\text{d}t}=\left(x\frac{\tau_{2}^{\tau_{1}/(\tau_{2}-\tau_{1})}}{\tau_{1}}-g\right)/\tau_{1},\\ \frac{\text{d}x}{\text{d}t}=-\frac{x}{\tau_{2}},\\ x\leftarrow x+w\text{ upon spike from synapse,} \end{array}$$

where *w* is a synaptic weight. We used τ_1_ = 0.5 ms and τ_2_ = 2.4 ms for *g*_AMPA_, τ_1_ = 4.0 ms and τ_2_ = 40.0 ms for *g*_NMDA_, and τ_1_ = 1.0 ms and τ_2_ = 7.0 ms for *g*_GABA_ based on existing studies (Hill and Tononi, 2005; Gerstner et al., 2014). In our study, the fourth-order Runge-Kutta method was used to calculate the neuron model.

### 2.4. STDP

We used the triplet rule of STDP (Pfister and Gerstner, 2006) to update the synaptic weights of excitatory connections. The triplet rule of STDP changes synaptic weights based on the firing timing of two postsynaptic spikes and one presynaptic spike, considering their firing frequency. The amount of change in a weight, Δ*w*, is determined using the following equations:


(7) $$\begin{array}{l} \text{Δ}w=\left\{ \begin{array}{l} -o_{1}(t)[A_{2}^{-}+A_{3}^{-}r(t-\epsilon )]\text{ if }t\text{ =}t^{\text{pre}},\\ r_{1}(t)[A_{2}^{+}+A_{3}^{+}o_{2}(t-\epsilon )]\text{ if }t\text{= }t^{\text{post}}, \end{array}\right.\; \end{array}$$

(8) $$\begin{array}{l} \begin{array}{c} \frac{\text{d}r_{1}\left(t\right)}{\text{d}t}=-\frac{r_{1}\left(t\right)}{\tau_{+}}\text{if }t=t^{\text{pre}},\text{then }r_{1}\leftarrow r_{1}+1,\\ \frac{\text{d}r_{2}\left(t\right)}{\text{d}t}=-\frac{r_{2}\left(t\right)}{\tau_{x}}\text{if }t=t^{\text{pre}},\text{then }r_{2}\leftarrow r_{2}+1,\\ \frac{\text{d}o_{1}\left(t\right)}{\text{d}t}=-\frac{o_{1}\left(t\right)}{\tau_{-}}\text{if }t=t^{\text{post}},\text{then }o_{1}\leftarrow o_{1}+1,\\ \frac{\text{d}o_{2}\left(t\right)}{\text{d}t}=-\frac{o_{2}\left(t\right)}{\tau_{y}}\text{if }t=t^{\text{post}},\text{then }o_{2}\leftarrow o_{2}+1, \end{array} \end{array}$$

where *t*_pre_ and *t*_post_ denote the presynaptic spike arrival time and the postsynaptic spike time, respectively. Synaptic weights increase and decrease with *t*_pre_ [long-time potentiation (LTP)] and *t*_post_ [long-time depression (LTD)], respectively. Here, $A_{2}^{+}$ and $A_{2}^{-}$ control the amplitudes of the weight change based on the two pair of spikes. The $A_{3}^{+}$ and $A_{3}^{-}$ control the amplitudes of LTP and LTD for the triplet rule, respectively. We used $A_{2}^{+}=5\times 10^{-11}$, $A_{2}^{-}=7\times 10^{-4}$, $A_{3}^{+}=6.2\times 10^{-4}$, and $A_{3}^{-}=2.3\times 10^{-5}$. The time constants τ_+_, τ_−_, τ_*x*_, and τ_*y*_ for controlling LTP and LTD decay were set as 16.8, 33.7, 101, and 125 ms, respectively. Accordingly, ϵ is a small positive constant that allows the weight to be updated prior to *r*_2_ or *o*_2_. Here, we used ϵ = 1 ms. These parameters were determined based on an existing study (Pfister and Gerstner, 2006). The weights of the inhibitory neurons (*W*_IE_ and *W*_II_) were not updated, and the minimum and maximum values of the weight were 0 and 0.04, respectively.

### 2.5. Analyses neural activity

Neural activity in each neuron group is represented as a time series of the LAP, which is the average membrane potential of excitatory neurons in a neuron group. Therefore, LAP reflects the synchronized neural activity of excitatory neurons. Here, we analyzed the MSE, MI, and TE of LAP signals to investigate the effect of local E/I imbalance in a neuron group on the complexity of neural activity and information transmission among neuron groups.

#### 2.5.1. Multiscale entropy

MSE, which indicates the complexity (degree of irregularity) of a time-series signal, is obtained by computing the sample entropy for a coarse-grained signal over multiple time scales (Costa et al., 2002, 2005). The procedure of calculation is as follows:

1. Downsample an original signal *x*(*t*) to obtain coarse-grained signal *y*(*t*) at scale ϵ:

(9) $$\begin{array}{l} y\left(t\right)=\frac{1}{\epsilon }\overset{i=t\epsilon }{\underset{i=\left(t-1\right)\epsilon +1}{\sum }}x\left(i\right)\;\left(1\leq t\leq N/\epsilon \right) \end{array}$$

2. Calculate sample entropy for each coarse-grained signal:

(10) $$\begin{array}{l} \text{SampEn}\left(r,m,N\right)=-\text{ln }\left[C_{m+1}\left(r\right)/C_{m}\left(r\right)\right], \end{array}$$

(11) $$\begin{array}{l} C_{m}\left(r\right)=\frac{\text{numberofpairs}\left(i,j\right)\left(|z_{i}^{m}-z_{j}^{m}|<r,i\neq j\right)}{\left(N-m+1\right)\left(N-m\right)}, \end{array}$$

where $z_{i}^{m}=\left\{y_{i},y_{i+1},\cdots \;,y_{i+m-1}\right\}$ represents a subsequence of the coarse-grained signals from the *i*th to the (*i* + *m* − 1)th data point of *y*(*t*), *m* denotes the length of the subsequence, *Y* = {*y*_1_, ⋯ , *y*_*i*_, ⋯ , *y*_*N*_} means the coarse-grained signals, and *N* denotes the length of *Y*.

We utilized LAP signal downsampled to 1 ms for each neuron group as *x*(*t*), and used *m* = 2 and *r* = 0.15, which are commonly used for MSE analysis.

#### 2.5.2. Information transmission

MI is a measure of mutual dependence between two time series. The MI between the LAP time series *x*_*i*_ of the *i*th neuron group and the external input signal *u* is calculated as:


(12) $$\begin{array}{l} \text{MI}\left(x_{i};u\right)=\underset{m\in x_{i}}{\sum }\underset{n\in u}{\sum }p\left(x_{i,m},u_{n}\right)\log\frac{p\left(x_{i,m}|u_{n}\right)}{p\left(x_{i,m}\right)}, \end{array}$$

TE is a measurement that determines how one time series affects another. The TE from the LAP time series *x*_*i*_ of the *i*th neuron group to the LAP time series *x*_*j*_ of the *j*th neuron group (where *i*≠*j*) is calculated as:


(13) $$\begin{array}{l} {\text{TE}}_{x_{i}\to x_{j}}=\underset{x_{j,t+1},x_{j}^{k},x_{i}^{l}}{\sum }p\left(x_{j,t+1},x_{j,t}^{k},x_{i,t}^{l}\right)\log\frac{p\left(x_{t+1}|x_{j,t}^{k},x_{i,t}^{l}\right)}{p\left(x_{j,t+1}|x_{j,t}^{k}\right)}, \end{array}$$

where, *l* and *k* indicate the historical lengths used to predict the future states and *t* denotes the current time step. In this study, we used *l* = 1 and *k* = 140 (= 7 ms). As TE includes the direction of the information flow, unlike MI, we can evaluate the information transmission from one neuron group receiving an external input to other neuron groups. We utilized Java Information Dynamics Toolkit (Lizier, 2014) to calculate MI and TE using the method developed by Kraskov, Stögbauer, and Grassberger (Kraskov et al., 2004).

## 3. Results

### 3.1. Two neuron groups

#### 3.1.1. STDP under increased E/I ratio strengthens intra- and interconnections

**Figure 2:**
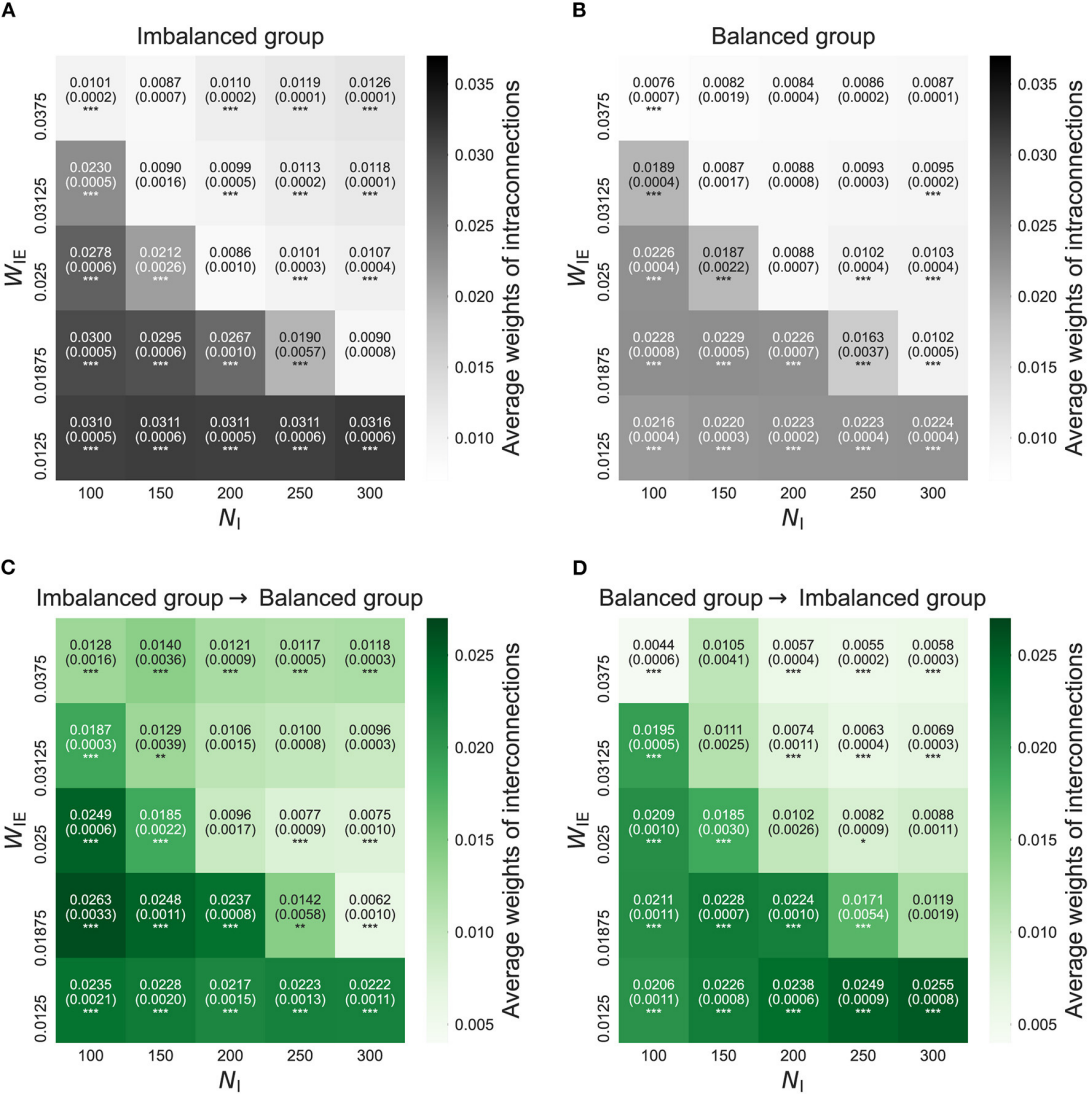
The average weights of intra- and interconnections after self-organization in the model with two neuron groups. **(A)** The average weights of intraconnections in the imbalanced neuron group. **(B)** The average weights of intraconnections in the balanced neuron group. **(C)** The average weights of interconnections from the imbalanced neuron group to the balanced neuron group. **(D)** The average weights of interconnections from the balanced neuron group to the imbalanced neuron group. The *x*- and *y*-axes show the number of inhibitory neurons (*N*_I_) and weights from inhibitory neurons to excitatory neurons (*W*_IE_) in the imbalanced neuron group, respectively. The number and the number in parentheses in each box represents the average and standard deviations of the weights among 20 simulations, respectively. ***p < 0.001, **p < 0.01, *p < 0.05 indicate statistical significance for differences compared to values for the model with base parameters (*N*_I_ = 200, *W*_IE_ = 0.025) using Welch's *t*-tests.

Figure 2 shows intra- and interconnections after the STDP process in the model with two neuron groups. The simulation result shows that the intra- and interconnections in both neuron groups, not only in the imbalanced neuron group, increased when the E/I ratio increased, i.e., weak inhibition. In the case of a decreased E/I ratio, i.e., strong inhibition, intra- and interconnections in the imbalanced neuron group slightly increased. However, intra- and interconnections in the balanced neuron group slightly decreased.

#### 3.1.2. Increased E/I ratio causes high firing rates

**Figure 3:**
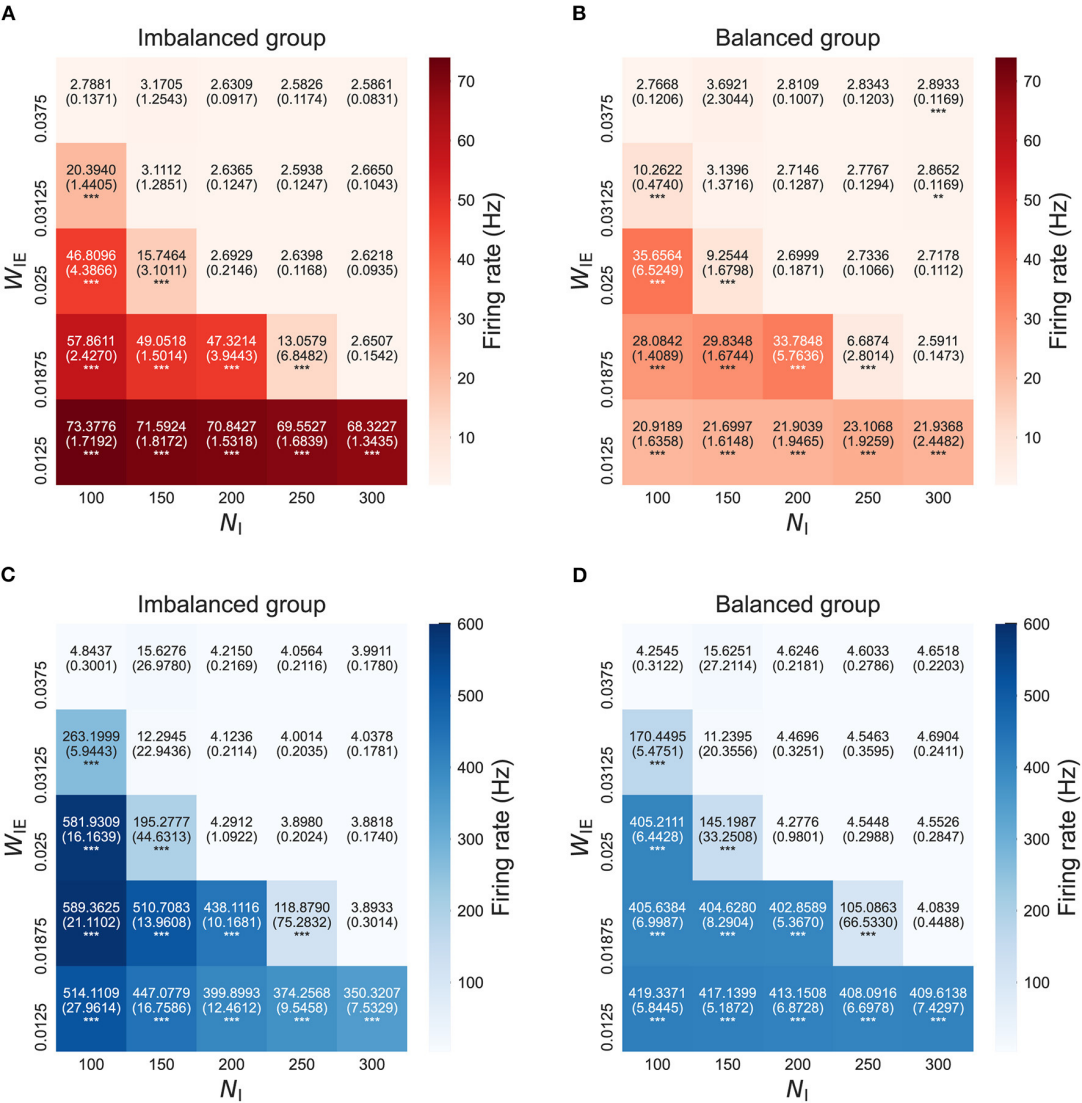
Firing rate after self-organization in the model with two neuron groups. **(A)** Firing rate of excitatory neurons in the imbalanced neuron group. **(B)** Firing rate of excitatory neurons in the balanced neuron group. **(C)** Firing rate of inhibitory neurons in the imbalanced neuron group. **(D)** Firing rate of inhibitory neurons in the balanced neuron group. The *x*- and *y*-axes show the number of inhibitory neurons (*N*_I_) and weights from inhibitory neurons to excitatory neurons (*W*_IE_) in the imbalanced neuron group, respectively. The number and the number in parentheses in each box represents the average and standard deviations of the firing rate among 20 simulations, respectively. ***p < 0.001, **p < 0.01 indicate statistical significance for differences compared to values for the model with base parameters (*N*_I_ = 200, *W*_IE_ = 0.025) using Welch's *t*-tests.

Figure 3 shows the firing rates of neuron groups after the STDP process in the model with two neuron groups. The results show that the firing rates in both neuron groups, not only in the imbalanced neuron group, increased with the increased E/I ratio. This finding might have been caused by increased intra- and interconnections, as shown in Figure 2.

#### 3.1.3. The complexity of neural activity decreases with the increased E/I ratio

**Figure 4:**
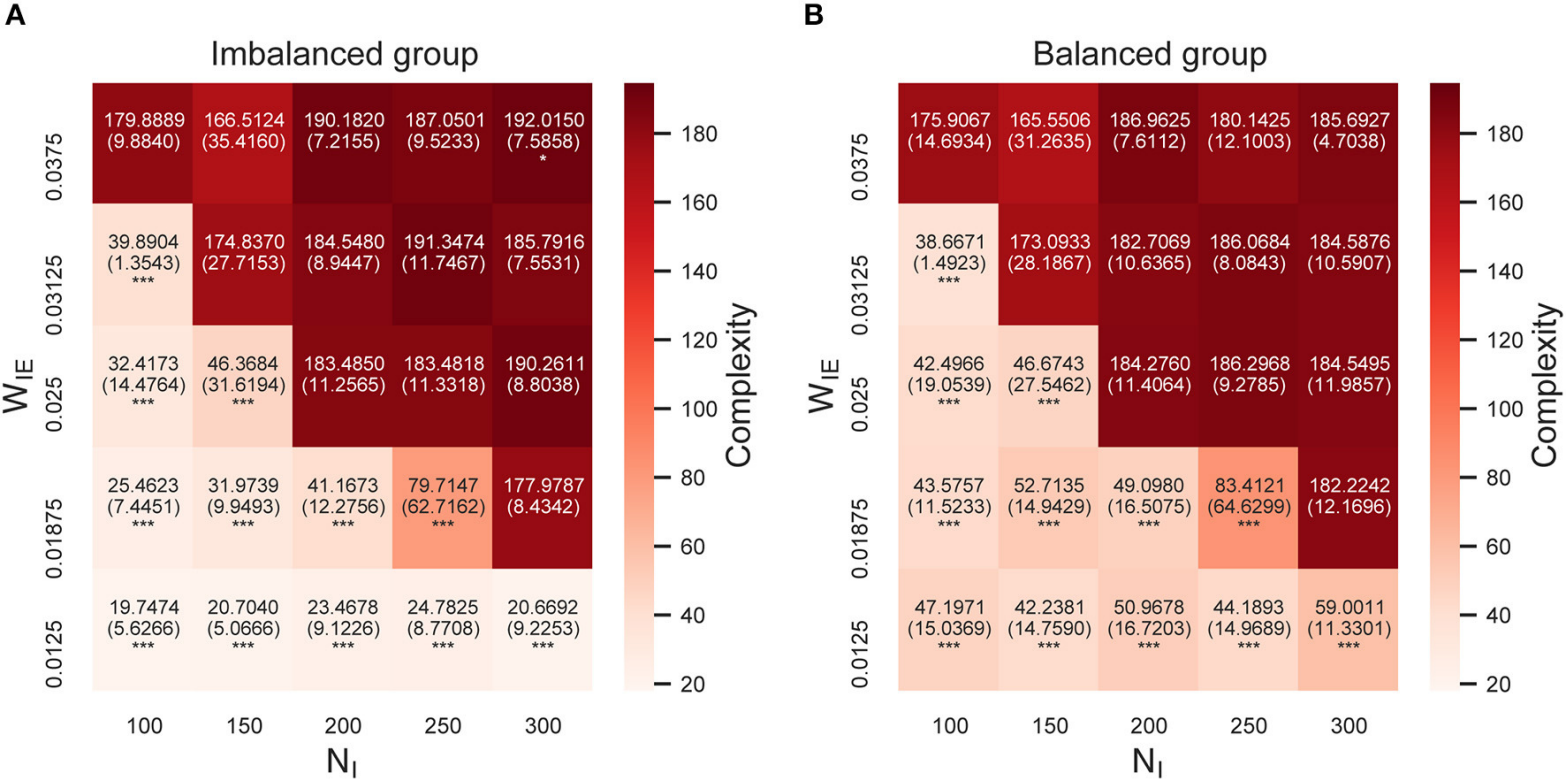
Complexity of neural activity after self-organization in the model with two neuron groups. **(A)** Complexity of neural activity in the imbalanced neuron group. **(B)** Complexity of neural activity in the balanced neuron group. The *x*- and *y*-axes show the number of inhibitory neurons (*N*_I_) and weights from inhibitory neurons to excitatory neurons (*W*_IE_) in the imbalanced neuron group, respectively. Color indicates the summation of the sample entropy for all 100 scale factors. The number and the number in parentheses in each box represents the average and standard deviations of the complexity among 20 simulations, respectively. ***p < 0.001, *p < 0.05 indicate statistical significance for differences compared to values for the model with base parameters (*N*_I_ = 200, *W*_IE_ = 0.025) using Welch's *t*-tests.

Figure 4 shows the complexity of neural activity (summation of sample entropy for 100 scale factors) after the STDP process in the model with two neuron groups. As shown in the figure, the complexity of neural activity in both neuron groups decreased when the E/I ratio increased. This result implies that the neural activity with low complexity in the imbalanced neuron group induced a decrease in the complexity of neural activity in the balanced neuron group through interconnections. Further, in the case of the increased E/I ratio, a decrease in the complexity of neural activity occurred in all scale factors, especially in the higher scale factors (see Supplementary Figure S1). Since the downsampling according to the scale factor acts like a low-pass filter, this result indicates a significant decrease in the complexity of neural activity in low-frequency bands with an increased E/I ratio.

The same analyses of neural activity in the model with two neuron groups without STDP showed lower firing rates and lower complexity than those with STDP, even in those with an imbalanced E/I ratio (see Supplementary Figures S2, S3). Further, the balanced neuron group was not affected by changes in the E/I ratio in the imbalanced neuron group. Thus, the relationship between the locally imbalanced E/I ratio and the altered complexity of neural activity/firing rates was induced by organization through STDP.

### 3.2. Ten neuron groups

To investigate the effect of the local E/I imbalance within a single neuron group on the whole network, we constructed a network model using ten neuron groups. We used a simple ring topology in which all neuron groups were coupled in the same way to eliminate the influence of network topology as much as possible and to clarify the graph-theoretical distance between neuron groups. Each neuron had interconnections with randomly selected neurons from four neighboring neuron groups. The results of only three cases ([high (H)-E/I; *N*_*I*_ = 100 and *W*_IE_ = 0.0125], [low (L)-E/I; *N*_*I*_ = 300 and *W*_IE_ = 0.0375], and [baseline (B)-E/I; *N*_*I*_ = 200 and *W*_IE_ = 0.025]) were given provided below to make the outcomes under different conditions comprehensible. An imbalanced neuron group with H-E/I and L-E/I had weak and strong inhibitions, respectively, compared with neuron groups with B-E/I.

### 3.2.1. STDP under locally increased E/I ratio induces over-connectivity in the network

**Figure 5:**
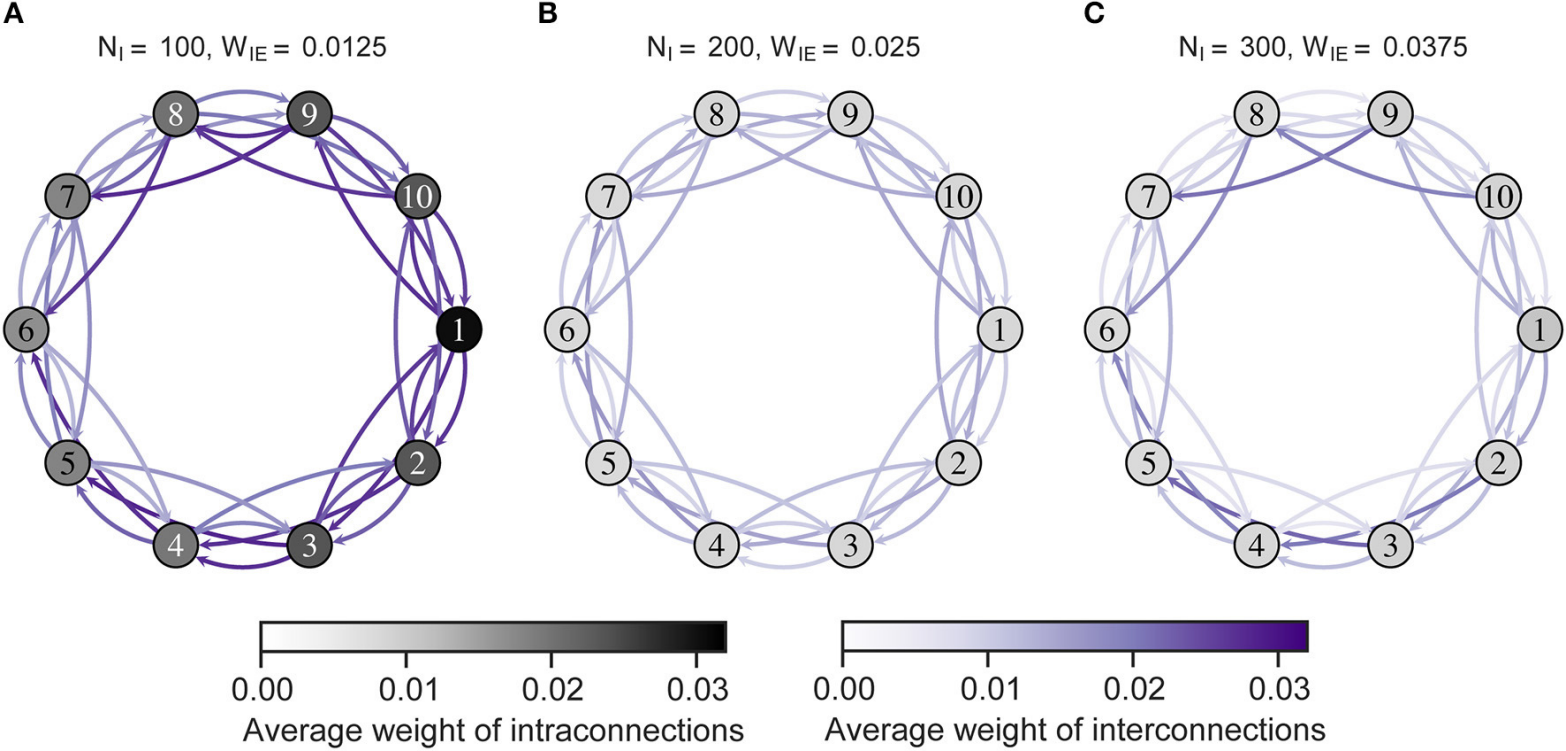
The average weights of intra- and interconnections after self-organization. Each numbered circle indicates a neuron group with its index. The color of the node indicates the average weights of intraconnections. Purple arrows indicate the average weights of interconnections from one neuron group to another. Neuron group 1 has an imbalanced E/I ratio. **(A)** High-E/I. **(B)** Base-E/I. **(C)** Low-E/I. E/I, excitation and inhibition; *W*_IE_, the inhibitory synaptic weights to excitatory neurons; *N*_I_, the number of inhibitory neurons.

Figure 5 shows intra- and interconnections after the STDP process in the model with ten neuron groups. The simulation results show that intra- and interconnections were greater in all neuron groups in H-E/I than those in L-E/I and B-E/I. That is, over-connectivity in the network was caused by locally weak inhibitory activity in one neuron group. Particularly, the intra- and interconnections around the imbalanced neuron group were stronger than other intra- and interconnections. In the case of L-E/I, interconnections between the imbalanced neuron group and other neuron groups slightly decreased compared to B-E/I, but several interconnections between balanced neuron groups slightly increased compared to B-E/I, e.g., interconnections between neuron groups from 3 to 5.

### 3.2.2. Low complexity of neural activity caused by locally increased E/I ratio in the network

**Figure 6:**
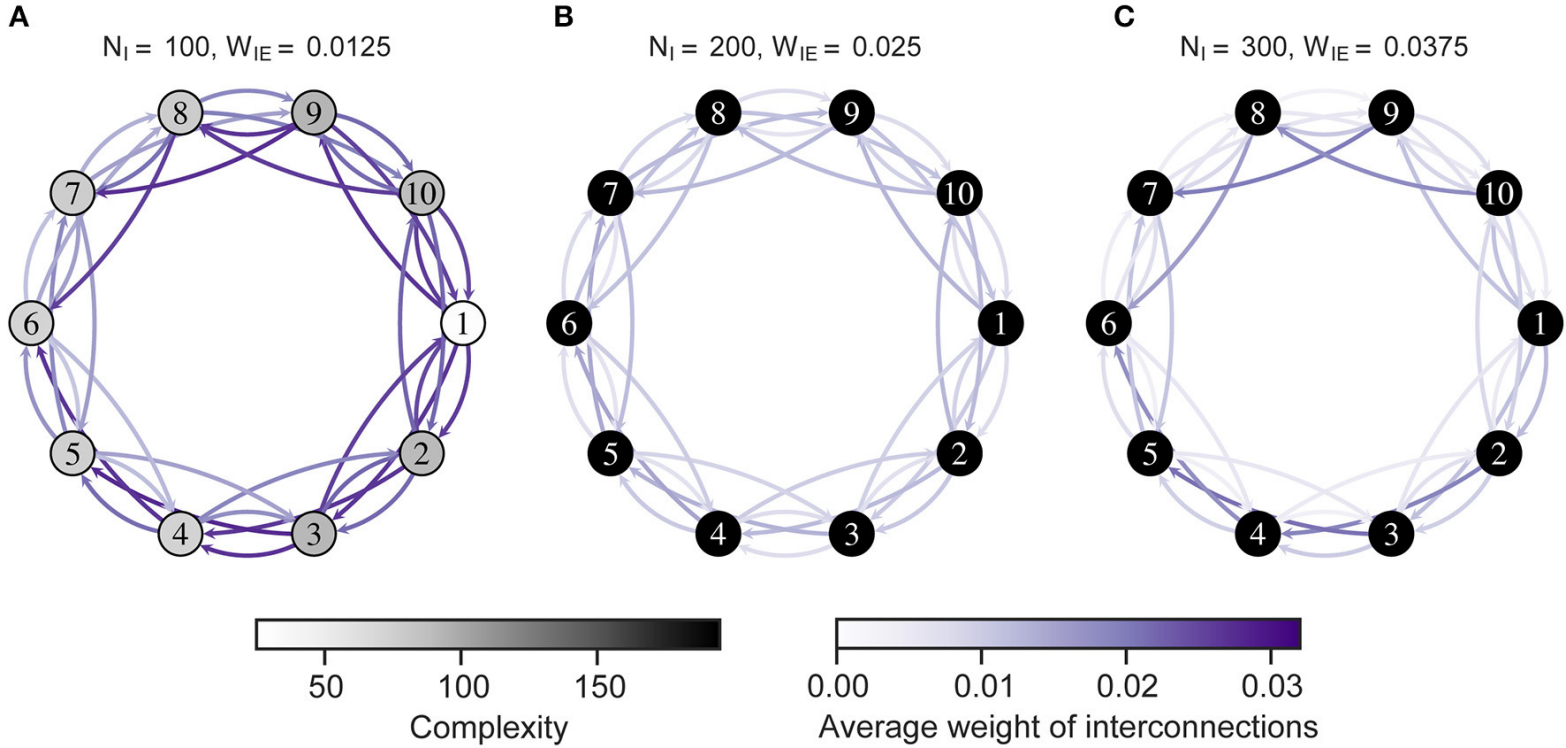
Complexity of neural activity in the neuron group after self-organization. Each numbered circle indicates a neuron group with its index. The color of the node indicates the complexity of neural activity in the neuron group that is the summation of the sample entropy for all 100 scale factors. Purple arrows indicate the average weights of interconnections from one neuron group to another. Neuron group 1 has an imbalanced E/I ratio. **(A)** High-E/I. **(B)** Baseline-E/I. **(C)** Low-E/I. E/I, excitation and inhibition; *W*_IE_, the inhibitory synaptic weights to excitatory neurons; *N*_I_, the number of inhibitory neurons.

Figure 6 shows the complexity of neural activity and interconnections after the STDP process. As shown in the figure, the complexity was low in all neuron groups in H-E/I. Notably, the neuron groups with low complexity corresponded to those with increased connectivity, as shown in Figure 5. Particularly, a neuron group with more incoming interconnections showed lower complexity than other groups (see Supplementary Figure S4). That is, the low complexity of neural activity in the network might result from excessive incoming connections that were strengthened by interactions with the imbalanced neuron group.

### 3.2.3. Locally increased E/I ratio globally disrupts the information transmission of the external input into the network

**Figure 7:**
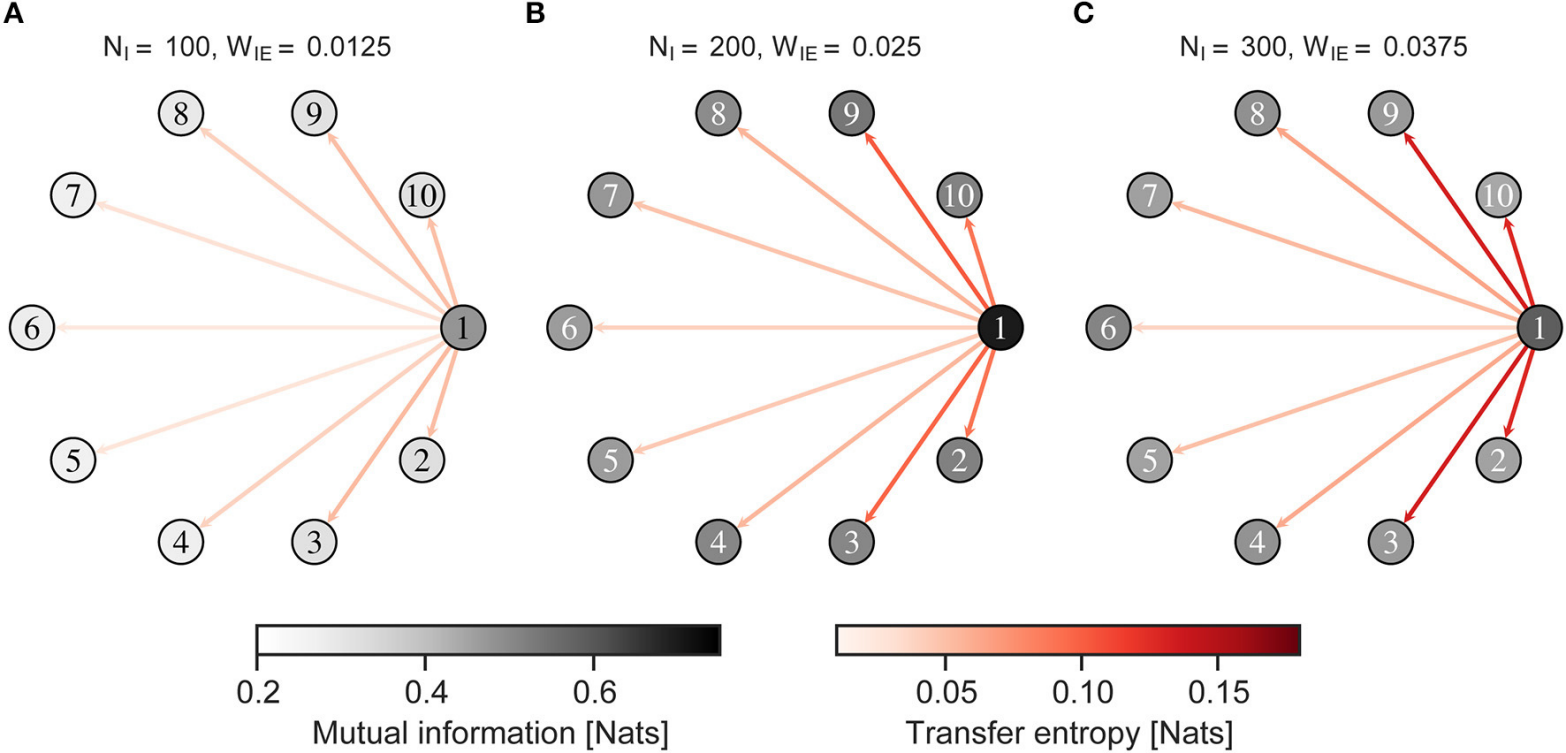
Mutual information and transfer entropy in the neuron group after self-organization. Each numbered circle indicates a neuron group with its index. The color of the node indicates the mutual information between neural activity of the neuron group and the external input. Red arrows indicate the transfer entropy from neural activity of one neuron group receiving external input to another. Neuron group 1 has an imbalanced E/I ratio. The external input was fed into neuron group 1. **(A)** High-E/I. **(B)** Baseline-E/I. **(C)** Low-E/I. E/I, excitation and inhibition; *W*_IE_, the inhibitory synaptic weights to excitatory neurons; *N*_I_, the number of inhibitory neurons.

**Figure 8:**
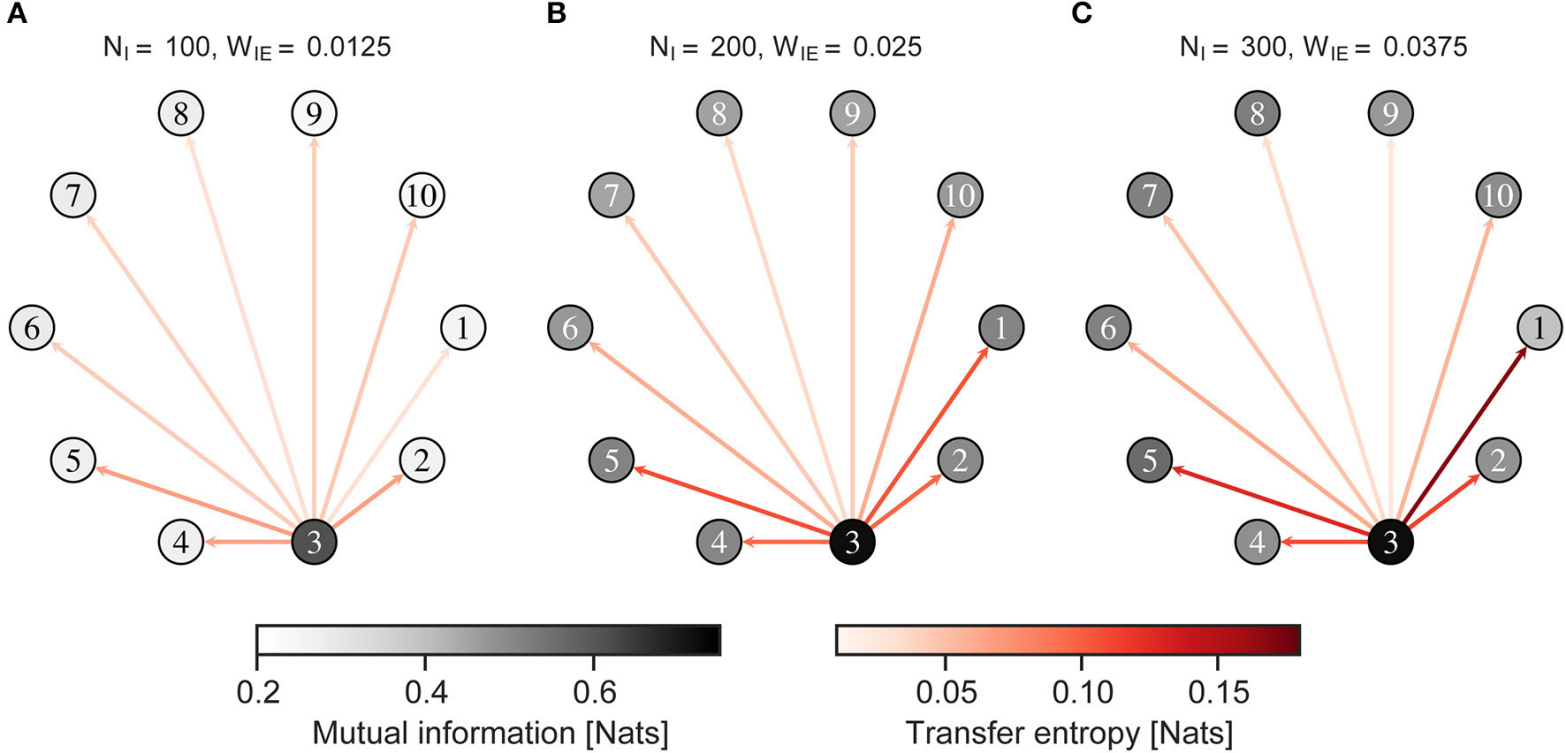
Mutual information and transfer entropy in the neuron group after self-organization. Each numbered circle indicates a neuron group with its index. The color of the node indicates the mutual information between neural activity of the neuron group and the external input. Red arrows indicate the transfer entropy from neural activity of one neuron group receiving external input to another. Neuron group 1 has an imbalanced E/I ratio. The external input was fed into neuron group 3. **(A)** High-E/I. **(B)** Baseline-E/I. **(C)** Low-E/I. E/I, excitation and inhibition; *W*_IE_, the inhibitory synaptic weights to excitatory neurons; *N*_I_, the number of inhibitory neurons.

Figure 7 shows the MI between the neural activity of the neuron group and external input, and the TE from neural activity of a neuron group receiving external input to those of other neuron groups, when the external input was given to an imbalanced group 1. Figure 8 shows almost the same, but the external input was given to a balanced neuron group 3. As shown in Figure 7, the MI and TE decreased in all neuron groups in H-E/I compared to those in other conditions. In the L-E/I condition, the TE was slightly higher than in the other conditions. However, when the external input was given to the balanced neuron group (Figure 8), the MI between the external input and neural activity of the imbalanced neuron group in L-E/I was slightly lower than that in the B-E/I condition (Tukey's test, *p* = 0.017). These results indicate that the imbalanced E/I ratio, especially the high E/I ratio, disrupted the information transmission of the external input to other neuron groups.

## 4. Discussion

In this study, we showed that organization under an increased E/I ratio in a neuron group induced excessive intra- and interconnections in neuron groups and decreased the complexity of neural activity. Notably, the results with ten neuron groups showed that a locally high E/I ratio within a single neuron group resulted in an increase in connections and decreased the complexity of neural activity in the entire network. In addition, the MI and TE decreased in the network in response to an external input. These phenomena were observed particularly when the inhibition was weak. If we suppose that the neuron group is a local brain region, these results can be interpreted that connections in the brain region become dense and the information transmission of an external input, e.g., sensory input, to other regions would become impaired. Our results suggest that physiological parameters concerning the E/I balance within a local area are important factors that support the interaction of brain structure and function through STDP and contribute to information transmission across the entire network.

### 4.1. Organization under the local E/I imbalance

Our results showed that an increased E/I ratio causes the decreased complexity of neural activity and increased intra- and interconnections. Based on these analyses, we consider a possible mechanism behind them as follows:

1. Since the imbalanced neuron group has weak inhibition, neurons are easily fired by synaptic input from the same neuron group or other neuron groups (see Supplementary Figures S2, S5).
2. STDP under the high-firing situation forms excessively strong intra- and interconnections (see Figures 2, 5; Supplementary Figure S6).
3. Strong intra- and interconnections increase synchronous firing of neurons (see Supplementary Figure S5). As a result, the complexity of neural activity decreases (see Figures 4, 6).
4. Excessive firing within the imbalanced neuron group also affects the other neuron groups through interconnections, resulting in increased connections and reduced complexity of neural activity in the network.

The STDP under synchronous neural activity in turn might strengthen the connections further by repeating the above procedures. Thus, structure-function interactions through STDP might magnify the effect of locally imbalanced E/I, leading to the global effect. This result is similar to our previous study (Park et al., 2019) that showed a decreased in the complexity of neural activity of neuron groups when many local connections were given. Therefore, our results showed that locally imbalanced E/I ratio induces local-over connectivity that reduces the complexity of neural activity.

### 4.2. Examining the relationship between our results and studies on neuropsychiatric disorders

Our results imply that the locally imbalanced E/I ratio might cause atypical brain structure and low complexity of brain activity, which are observed in neuropsychiatric disorders. As shown in Figures 2, 5, intra- and interconnections increased with an increased E/I ratio. These results are similar to those reported by ASD and schizophrenia studies, which showed increased neural density within certain local areas (Selemon et al., 1998; Casanova et al., 2006) and excessive interconnections (Li et al., 2014; Solso et al., 2016), respectively. Our MSE analysis showed the complexity of neural activity decreased with an increased E/I ratio (Figure 4), especially at high scales (Supplementary Figure S1). This result aligns with those of studies that showed lower complexity of functional near-infrared spectroscopy (Xu et al., 2020), EEG (Bosl et al., 2011), and MEG (Ghanbari et al., 2015; Hadoush et al., 2019) signals in children with ASD compared to that of TD children or children with mild ASD. Further, similar to our previous studies (Park et al., 2019), the current simulation results showed a decrease in the complexity of neural activity of neuron groups with many local connections, especially incoming interconnections (Figure 6; Supplementary Figure S5). Thus, our model could predict that a decrease in the complexity of brain activity in a brain region of ASD or schizophrenia might be induced by excessive synaptic input from other brain regions through incoming interconnections that were strengthened by interactions with the brain region with imbalanced E/I ratio.

However, in contrast to studies showing increased interconnections that are consistent with our results, weak interconnections with distant regions have also been reported in ASD (Rane et al., 2015). Ghanbari et al. (2015) showed that although the complexity of brain activity in children with ASD compared with children with TD was lower in some regions (frontal regions in the delta band and occipital-parietal regions in the alpha band), it was higher in other regions (parietal regions in delta, central and temporal regions in theta, and frontal-central boundary regions in gamma). Furthermore, even though our results showed that an increase in the E/I ratio increased the firing rate and connections, studies of ASD and Schizophrenia using fMRI or positron emissions tomography showed hyper- and hypoactivity during resting state appeared simultaneously depending on regions (e.g., hyperactivity in the right supplementary motor area and bilateral lingual gyrus, and hypoactivity in the right middle temporal gyrus and ventromedial prefrontal cortex) (Kuhn and Gallinat, 2013; Wang et al., 2018). We associate these differences to the effect of different structures in each region. In our study, we assumed that all neuron groups have the same types of neurons and connections (each neuron group is regularly connected with neighboring neuron groups), but the human brain has different types of neurons and long-range connections with other regions, depending on the region. Especially, existing computational studies showed the a neuron group with many interconnections decreases the complexity of the neural activity (Park et al., 2019), and small-world network structure suppresses the chaoticity in neural activity (Kawai et al., 2019). Future studies using a computational model with various network structures, e.g., small-world network, scale-free network, or network structure based on real human connectome data, and neuron types may clarify our speculation.

Many studies reported hypersensitivity or hyposensitivity to external input in ASD (Hazen et al., 2014; Schauder and Bennetto, 2016) and dysfunction of sensory processing in schizophrenia (Javitt and Sweet, 2015). We found that the locally increased E/I ratio decreased information transmission in the entire network (see Figures 7, 8). These results suggest a possibility that abnormal neural responses to external input in ASD and schizophrenia might originate from an imbalanced E/I ratio in not only in sensory area but also in other regions.

### 4.3. Limitations of the study and future work

The current model had fixed control parameters with regard to inhibition, and it had plastic excitatory synapses that were updated according to STDP. However, several physiological studies have shown that inhibitory synapses also exhibit plasticity (Caporale and Dan, 2008). A neurophysiological study using rats (D'amour and Froemke, 2015) and computational studies (Vogels et al., 2011; Akil et al., 2021) showed that STDP of inhibitory synapses contributes to homeostasis of neural activity. Especially, Wang and Maffei (2014) showed that excitatory LTP decreases as inhibitory LTP increases in the visual cortex of rats. Intrinsic plasticity that changes the intrinsic electrical properties of neurons is another mechanism that directly contributes to homeostasis (Turrigiano, 2011). If we consider synaptic and intrinsic homeostatic plasticity, the actual effects of the E/I balance might be less than those in our study. In addition, the time window for LTP and LTD of excitatory plasticity or inhibitory plasticity differs depending on brain regions and layers (Caporale and Dan, 2008). In the future, the relationship between several types of plasticity, including homeostatic plasticity, and E/I balance should be investigated in detail. Further, we only used regular-spiking neurons and fast-spiking neurons for excitatory neurons and inhibitory neurons, respectively. Although these neurons occupy a large proportion of the cortex, other types of neurons, bursting type neurons or somatostatin-expressing neurons, also exist, and their importance in neural dynamics has been discussed (Tremblay et al., 2016; Zeldenrust et al., 2018). The contribution of different types of inhibitory neurons to the information transmission in the organized network is also a fascinating subject.

Although we evaluated the complexity and information transmission of neural activity, we did not assess how the transferred information and the complexity of neural activity contribute to cognitive functions and motor behavior. Moreover, in this study, organization of the network occurred with Poisson input. However, the brains of humans and animals receive structured sensory signals through the body and reflect them in behavior. The mechanism through which altered E/I balance affects the self-organization of the brain and behavior through interactions with the environment is a cutting-edge research topic in developmental science, especially in the field of developmental disorders.

## Data availability statement

The raw data supporting the conclusions of this article will be made available by the authors, without undue reservation.

## Author contributions

JP and YK conceived and designed the research and interpreted the data. JP developed software and simulations, performed experiments, collected data, analyzed data, and drafted the manuscript. JP, YK, and MA reviewed and edited the manuscript. All authors contributed to the article and approved the submitted version.
