# Supplementary material for: Spike timing-dependent plasticity under imbalanced excitation and inhibition reduces the complexity of neural activity
Source: Front Comput Neurosci. 2023 Apr 12;17:1169288. doi: 10.3389/fncom.2023.1169288 (PMC10130424; doi:10.3389/fncom.2023.1169288)
Supplement: Supplementary file 1 [file Data_Sheet_1.pdf]

Supplementary Material

SUPPLEMENTARY FIGURES

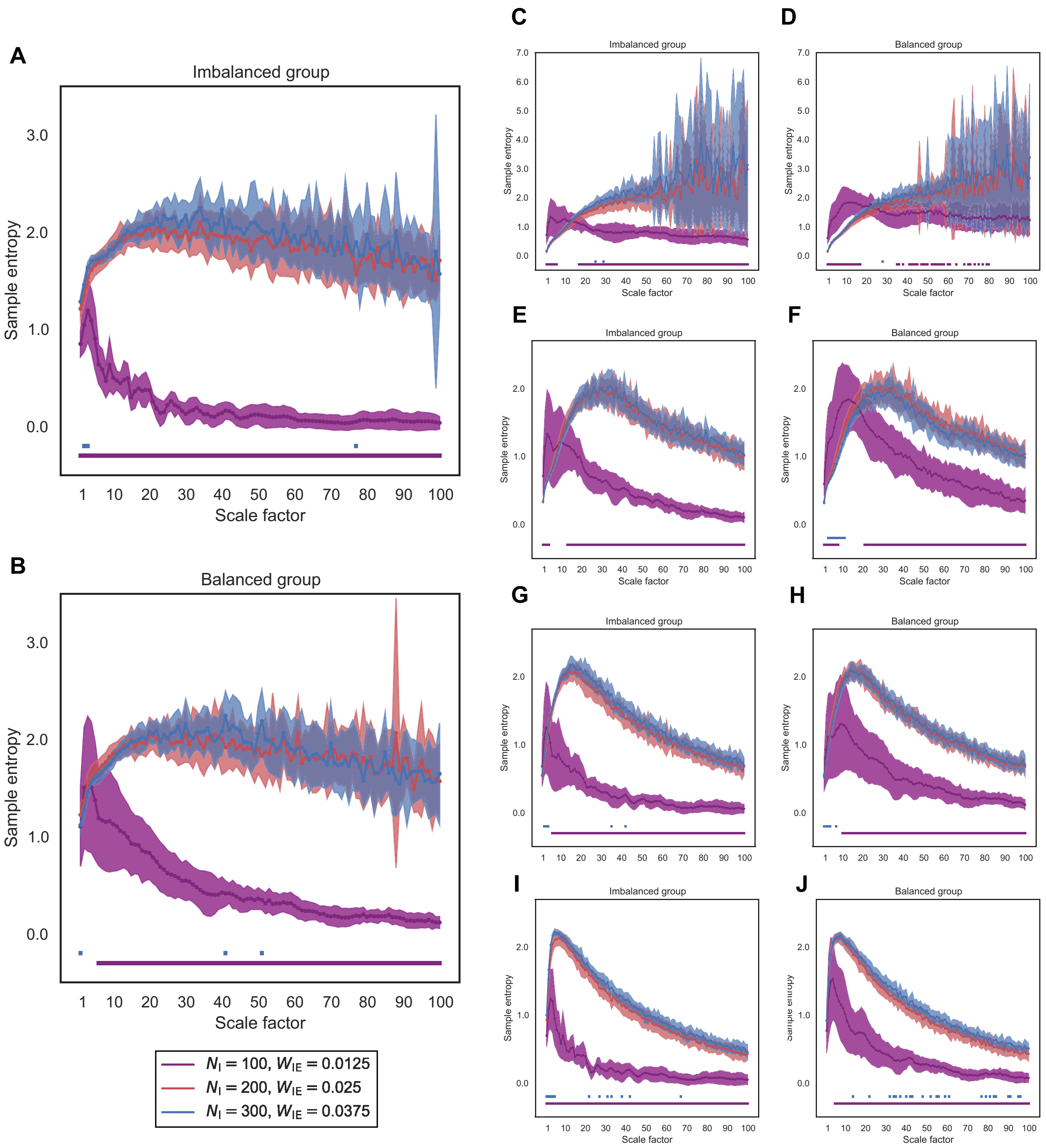

Figure S1. Multiscale entropy (MSE)-based complexity curves at each frequency for each neuron group in a model with two neuron groups. (A) Sample entropy in the imbalanced neuron group. (B) Sample entropy in the balanced neuron group. (C) Sample entropy in the imbalanced neuron group (delta band (1-4 Hz)). (D) Sample entropy in the balanced neuron group (delta band (1-4 Hz)). (E) Sample entropy in the imbalanced neuron group (alpha band (8-12 Hz)). (F) Sample entropy in the balanced neuron group (alpha band (8-12 Hz)). (G) Sample entropy in the imbalanced neuron group (beta band (14-30 Hz)). (H) Sample entropy in the balanced neuron group (beta band (14-30 Hz). (I) Sample entropy in the imbalanced neuron group (gamma band (30-80 Hz)). (J) Sample entropy in the balanced neuron group (gamma band (30-80 HZ). Color curves and color-shaded areas represent average and standard deviation values among 20 simulations, respectively. The x-axis indicates the scale factor, and the y-axis indicates the sample entropy. The color lines under the color curve indicate scales that are statistically signifiant ($p < 0.001$) compared to values for the model with base parameters ($N_{\mathrm{I}} = 200$, $W_{\mathrm{IE}} = 0.025$) using Welch's t-tests.

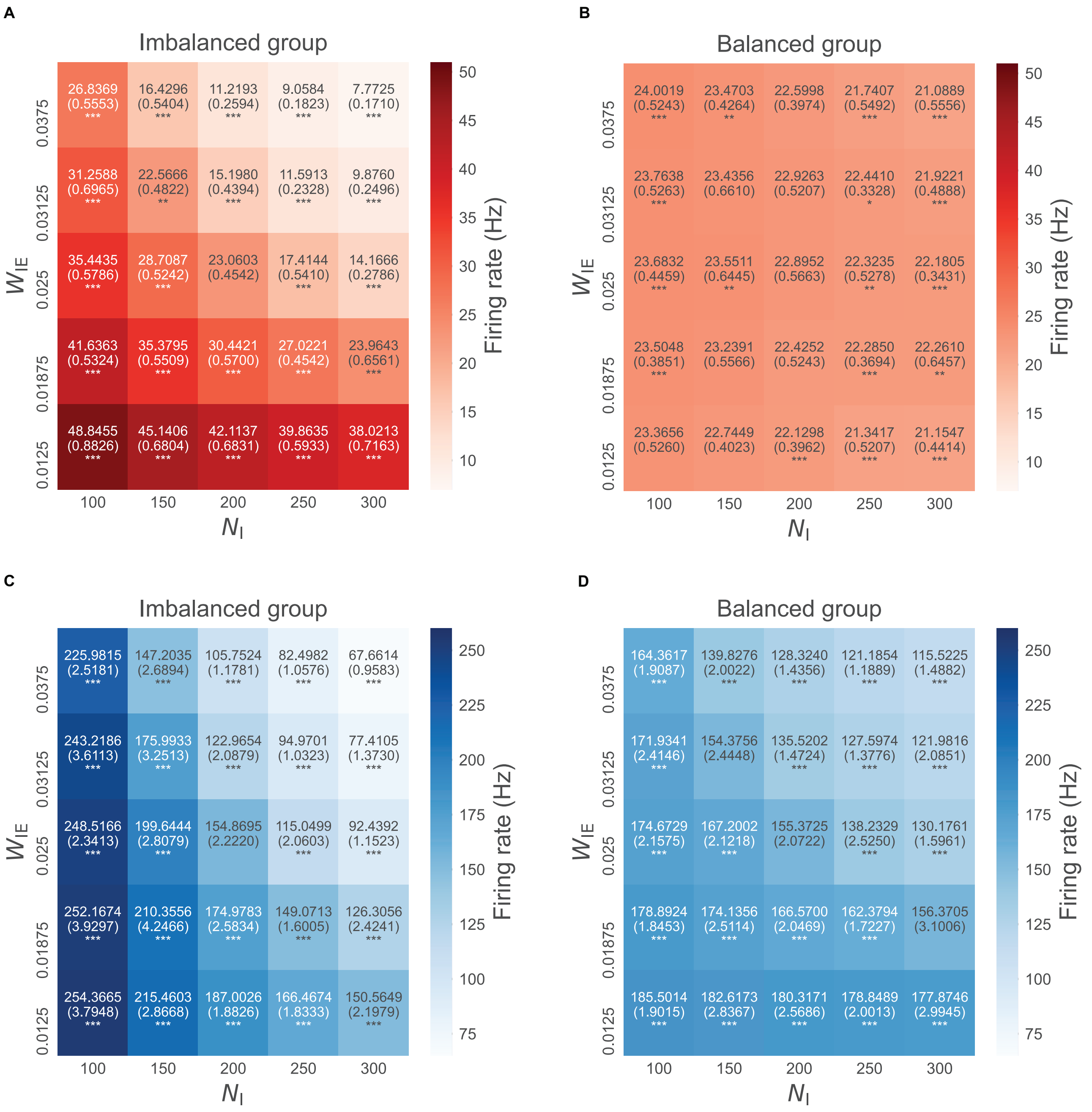

Figure S2. The firing rate of neurons without STDP in the model with two neuron groups. (A) The firing rate of excitatory neurons in the imbalanced neuron group. (B) The firing rate of excitatory neurons in the balanced neuron group. (C) The firing rate of inhibitory neurons in the imbalanced neuron group.(D) The firing rate of inhibitory neurons in the balanced neuron group. The x- and y-axes show the number of inhibitory neurons (N_I) and weights from inhibitory neurons to excitatory neurons (W_IE) in the imbalanced neuron group, respectively. The number and the number in parentheses in each box represents the average and standard deviations of the weights among 20 simulations, respectively. *** $p < 0.001$, ** $p < 0.01$, * $p < 0.05$ indicate statistical significance for differences compared to values for the model with base parameters ($N_\mathrm{I} = 200$, $W_\mathrm{IE} = 0.025$) using Welch's t-tests.

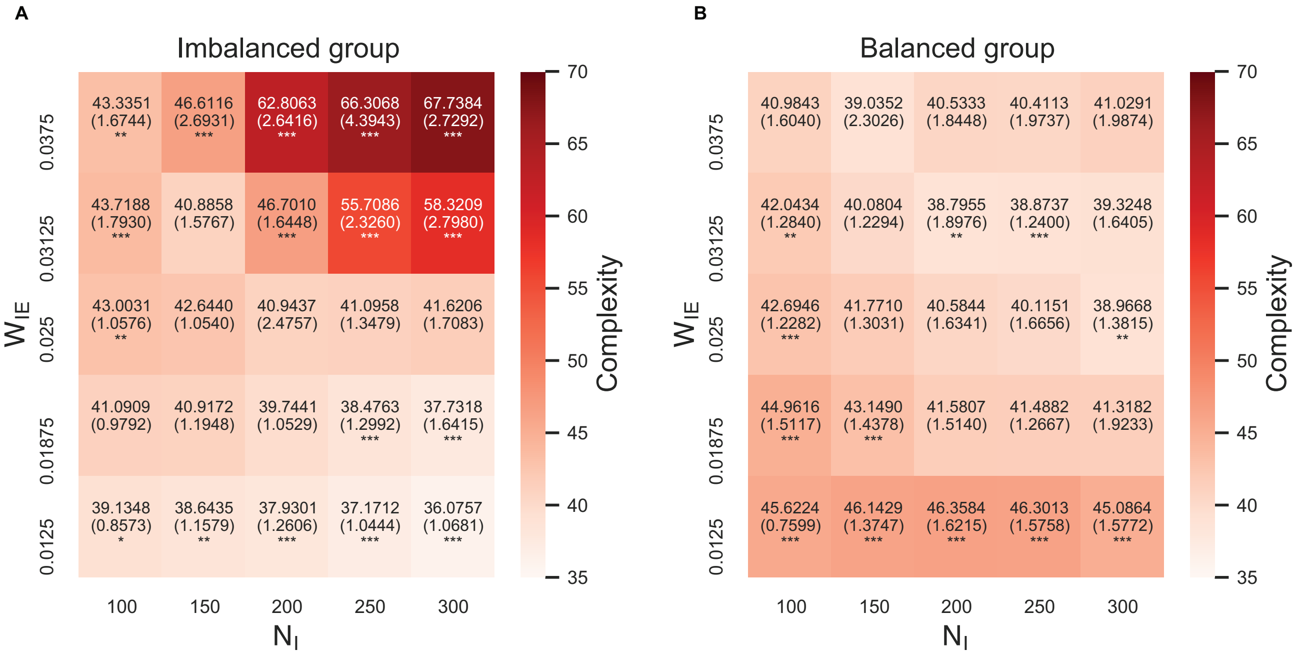

Figure S3. Complexity of neural activity without STDP in the model with two neuron groups. (A) Complexity of neural activity in the imbalanced neuron group. (B) Complexity of neural activity in the balanced neuron group. The x- and y-axes show the number of inhibitory neurons (N_I) and weights from inhibitory neurons to excitatory neurons (W_IE) in the imbalanced neuron group, respectively. The color indicates the summation of the sample entropy for all 100 scale factors. The number and the number in parentheses in each box represent the average and standard deviations of the weights among 20 simulations, respectively. *** $p < 0.001$, ** $p < 0.01$, * $p < 0.05$ indicate statistical significance for differences compared to values for the model with base parameters ($N_\mathrm{I} = 200$, $W_\mathrm{IE} = 0.025$) using Welch's t-tests.

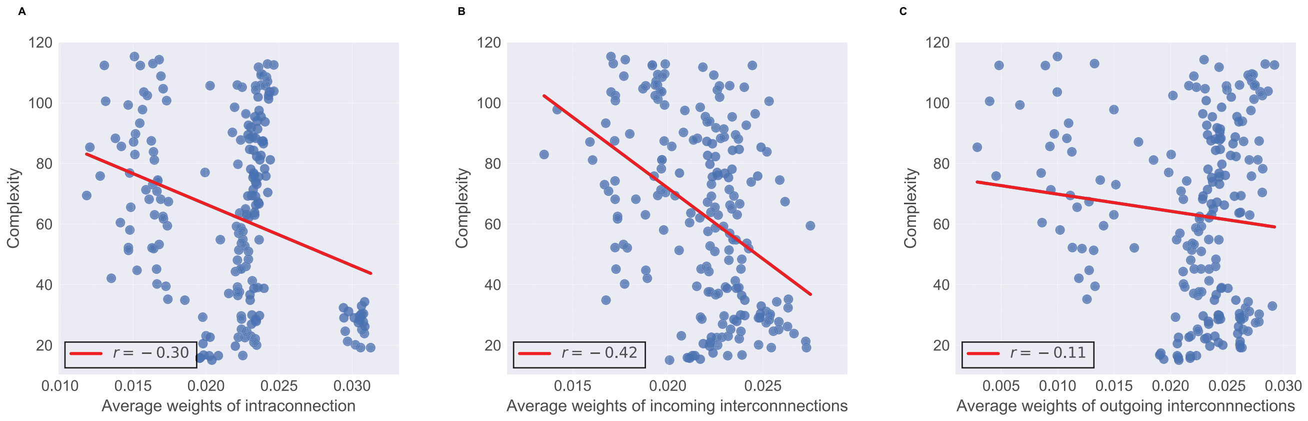

Figure S4. Relationship between connections of a neuron group and the complexity after self-organization in High-E/I. (A) Relationship between intraconnections and the complexity (The p-value of Pearson correlation coefficient is 1.82-05). (B) Relationship between incoming interconnections and the complexity (The p-value is 5.08e-10). (C) Relationship between outgoing interconnections and the complexity (The p-value is 0.13). The x- and y-axes show average weights of incoming interconnections and the complexity of neural activity, respectively. Each marker corresponds to a neuron group. The red line indicates correlation.

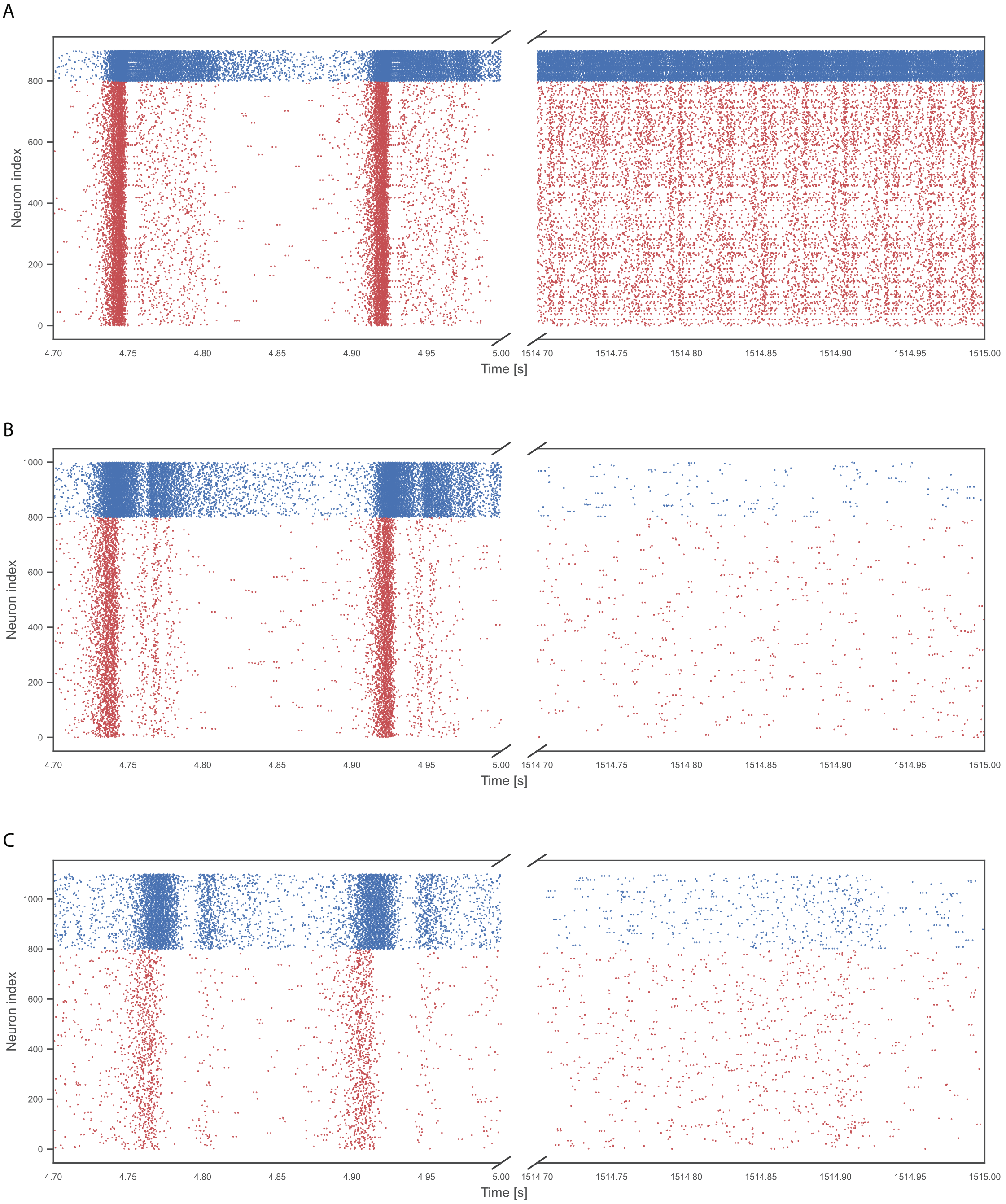

Figure S5. Raster plot of the imbalanced neuron group before and after self-organization in the model with two neuron groups. (A) $N_{\mathrm{I}} = 100$, $W_{\mathrm{IE}} = 0.0125$. (B) $N_{\mathrm{I}} = 200$, $W_{\mathrm{IE}} = 0.025$. (C) $N_{\mathrm{I}} = 300$, $W_{\mathrm{IE}} = 0.0375$. Red dots and blue dots in the raster plot represent spike of excitatory neurons and inhibitory neurons, respectively. The x-axis indicates the time, and the y-axis indicates the neuron index.

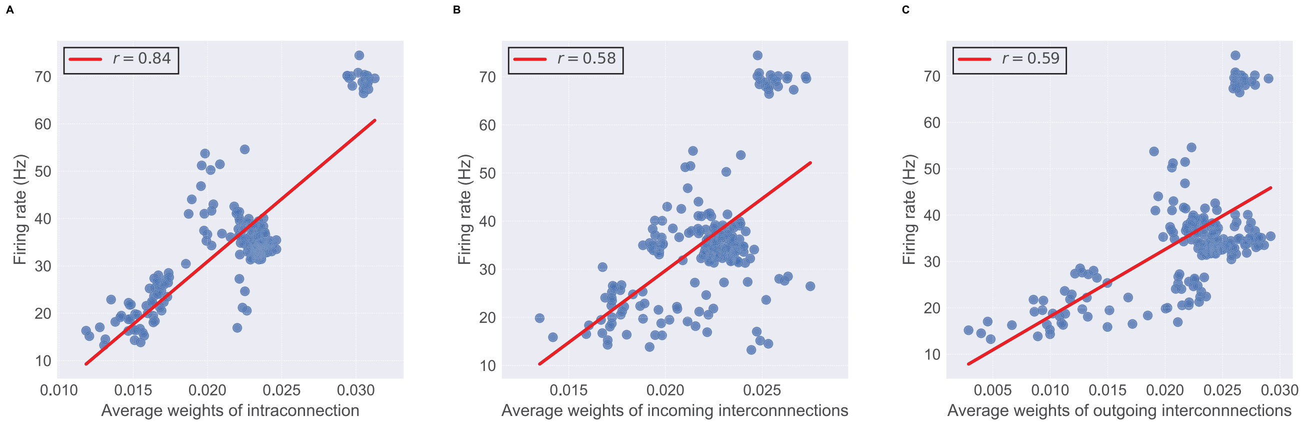

Figure S6. Relationship between connections of a neuron group and the firing rate of excitatory neurons after the self-organization in High-E/I. (A) Relationship between intraconnections and the firing rate of excitatory neurons (The p-value of Pearson correlation coefficient is 1.28e-55). (B) Relationship between incoming interconnections and the firing rate of excitatory neurons (The p-value is 1.21e-19).(C) Relationship between outgoing interconnections and the firing rate of excitatory neurons (The p-value is 4.75e-20). Relationship between incoming interconnections of a neuron group and the complexity after the self-organization in High-E/I. The x- and y-axes show average weights of incoming interconnections and the firing rate of of excitatory neurons, respectively. Each marker corresponds to a neuron group. The red line indicates correlation.
